# A Nurse’s Hand-Book of Medicine

**Published:** 1906-08

**Authors:** 


A NURSE'S HAND-BOOK OF MEDICINE. By J. Norman Henry, M.D., Clinical Professor of Medicine, Woman's Medical College of Pennsylvania, etc. J. B. Lippincott Co., Publishers, Philadelphia and London.

This little book comprises the substance of lectures delivered by Dr. Henry before the nurses of the Pennsylvania Hospital during the last four years, and is devoted to general medical information for nurses and those interested in nursing. The common diseases are briefly and simply discussed, and the significance of variations in the excretions, temperature, pulse, respiration, are given.

Chapter V goes into the personal hygiene, hygiene of the sick-room, care of contagious diseases and disinfection in a practical manner. Dr. Henry urges that women shall thoroughly study the disagreeable as well as the brighter side of nursing before adopting this arduous vocation, and that their minds should be disabused of the sentimental idea of the trained nurse, which prompts many unfit to enter training schools.

